# Host Age Affects the Development of Southern Catfish Gut Bacterial Community Divergent From That in the Food and Rearing Water

**DOI:** 10.3389/fmicb.2018.00495

**Published:** 2018-03-20

**Authors:** Zhimin Zhang, Dapeng Li, Mohamed M. Refaey, Weitong Xu, Rong Tang, Li Li

**Affiliations:** ^1^Department of Fishery Resources and Environment, College of Fisheries, Huazhong Agricultural University, Wuhan, China; ^2^Hubei Provincial Engineering Laboratory for Pond Aquaculture, Wuhan, China; ^3^Department of Animal Production, Faculty of Agriculture, Mansoura University, Al-Mansoura, Egypt

**Keywords:** host age, fish, gut microbiota, microbial ontogenesis, rearing water, food

## Abstract

Host development influences gut microbial assemblies that may be confounded partly by dietary shifts and the changing environmental microbiota during ontogenesis. However, little is known about microbial colonization by excluding dietary effects and compositional differences in microbiota between the gut and environment at different ontogenetic stages. Herein, a developmental gut microbial experiment under controlled laboratory conditions was conducted with carnivorous southern catfish *Silurus meridionalis* fed on an identical prey with commensal and abundant microbiota. In this study, we provided a long-term analysis of gut microbiota associated with host age at 8, 18, 35, 65, and 125 day post-fertilization (dpf) and explored microbial relationships among host, food and water environment at 8, 35, and 125 dpf. The results showed that gut microbial diversity in southern catfish tended to increase linearly as host aged. Gut microbiota underwent significant temporal shifts despite similar microbial communities in food and rearing water during the host development and dramatically differed from the environmental microbiota. At the compositional abundance, *Tenericute*s and *Fusobacteria* were enriched in the gut and markedly varied with host age, whereas *Spirochaetes* and *Bacteroidetes* detected were persistently the most abundant phyla in food and water, respectively. In addition to alterations in individual microbial taxa, the individual differences in gut microbiota were at a lower level at the early stages than at the late stages and in which gut microbiota reached a stable status, suggesting the course of microbial successions. These results indicate that host development fundamentally shapes a key transition in microbial community structure, which is independent of dietary effects. In addition, the dominant taxa residing in the gut do not share their niche habitats with the abundant microbiota in the surrounding environment. It's inferred that complex gut microbiota could not be simple reflections of environmental microbiota. The knowledge enhances the understanding of gut microbial establishment in the developing fish and provides a useful resource for such studies of fish- or egg-associated microbiota in aquaculture.

## Introduction

All animals start to host complex gut bacterial communities since their hatching or birth. Gut bacterial community differed among individuals or populations (Salonen et al., 2014; Waite et al., 2014; Hill et al., 2017). In humans, gut microbial colonization of infants is related to delivery modes associated with environmental microbiota and type of feeding (breast/formula) (Dominguez-Bello et al., 2010; Van et al., 2015; Rutayisire et al., 2016; Laursen et al., 2017). The process of microbial colonization develops rapidly and varies greatly during the development (Bäckhed et al., 2015; Lim et al., 2015; Tarnecki et al., 2016). The early consequences directly involve infant gut function and immune system, and they are even associated with the signatures of the late gut microbiota as well as health and fitness of a host. These findings, therefore, give rise to great interest in gut microbiota of ontogenetic development in vertebrates such as birds, broilers, pig, and fish (Jami et al., 2013; Waite et al., 2014; Bakke et al., 2015; Corrigan et al., 2015; Niu et al., 2015; Best et al., 2016).

Fish directly interact with microbiota in the surrounding environment including both water and food. Early research on emergences of the microbiota in larval fish gut revealed microbial transfer from the external environment to gut with rapid microbial colonization (Hansen and Olafsen, 1999). The degree of external microbial colonization in the gut is related to environmental origins (Bakke et al., 2013; Giatsis et al., 2015). Gut microbiota at the early life stages of host largely differs from microbial communities present in diet-supply and/or surrounding water (Bledsoe et al., 2016; Schmidt et al., 2016). Although many lines of evidence obtained from adult individual species have revealed that the succession of gut microbial consortia is modulated by a wealth of deterministic processes and stochastic processes (Burns et al., 2016; Llewellyn et al., 2016; Yan et al., 2016; Vega and Gore, 2017), it is difficult to conclude that which process or perturbation at different life stages the establishments and alterations of gut microbial communities depend on.

In recent years, some studies focus on early developmental gut microbiota in fish. Specifically, the studies of a model organism, zebrafish, (*Danio rerio*) (Jemielita et al., 2014; Burns et al., 2016; Zac Stephens et al., 2016) as well as several commercial species are performed such as cod (*Gadus morhua*) (Bakke et al., 2015), channel catfish (*Ictalurus punctatus*) (Bledsoe et al., 2016) and gibel carp (*Carassius auratus gibelio*) (Li et al., 2017). The previous studies consistently indicate that the gut microbial assemblages change over time. But it should be noted that the changes are possibly related to different food supplies to the hosts during their development. Therefore the process of gut microbial assemblages is likely to be affected by diet shifts, for example from live feeds to commercial feeds, and even affected by interactive effects between host age and diet (Bakke et al., 2015; Bledsoe et al., 2016; Li et al., 2017).

Due to the restrictions on early food resource availability and gut development, it is unavoidable for newly hatched individuals to be faced with different food resources over ontogenesis. This phenomenon is common to larva culture of commercial fish species which are usually fed on live feeds as transitional foods, such as water earthworm, fairy shrimp, copepod, rotifer or artemia, and subsequently the diets were switched to commercial feeds (Bakke et al., 2015; Bledsoe et al., 2016; Li et al., 2017). One recent experiment was designed to overcome age-related dietary effects by Wong et al. (2015) who used developing zebrafish fed on one artificial diet on long-term basis to explore age-specific gut microbial assemblages. It is necessary to disentangle mutualistic interactions between ontogenesis and gut microbial colonization with controllable exogenous perturbations. However, the use of sterile custom diet limits the exploration of potential microbial interactions between gut and diet in the context of host development. In addition, whether and how the consumed food microbiota corresponds with a microbial community in the gut remains poorly understood. Given that microbiota naturally inhabits in live feeds might interact with the host, it is of importance to understand the way in which early gut acquire microbiota and to reveal the microbial changes at different developmental stages.

Southern catfish (*Silurus meridionalis*) is an important freshwater carnivorous fish species. This species has no muscular bones and grows rapidly with about a year of culture cycle on a commercial scale. The catfish are fed on small fish or fresh prey items in aquaculture, which makes it an ideal species to study microbial assemblages in the gut associated with microbiota in their surrounding environment (including both food and rearing water) during the ontogenesis. This study was designed to explore ontogenetic patterns of the gut microbial structure of southern catfish. In addition to addressing gut microbiota in southern catfish fed on an identical food source under rigorously controlled conditions (such as the same parents, the single food and the same culture system) in the course of 125-day developmental ontogeny, we further explored the underlying assemblages mechanisms by comparing microbiota residing in the gut to that in the environment.

## Materials and methods

### Experimental animals and protocol

Two breeding stock southern catfish (a male and a female) that feed on their cohousing small fish (several carps) were collected from a pond and then used for offspring reproduction. Fertilized eggs and a feed used in this study were provided by a local commercial southern catfish farm. The experimental protocols were approved by the Animal Ethics Committee of the Huazhong Agricultural University, China under permit number HZAUMO-2016-026.

The fertilized eggs were transported to an incubator in College of Fisheries, Huazhong Agricultural University (Wuhan, China), and hatched at a density of about 18 eggs/L aerated tap water in a flowing water system during a natural light period. After ~2 dpf, sac-fry larvae were transported to two independent flow-through tanks (~1000 fish/tank) for subsequent culture experiment in which fish were fed with minced water earthworm to apparent satiation at 4~5 dpf and then fed with untreated water earthworm from 7 dpf to the end of the experiment (four times daily at 07:00, 13:00, 19:00, and 24:00 before 35 dpf and three times daily at 07:00, 16:00, and 22:00 after 35 dpf). In addition to automatic flowing water exchange, the tanks were regularly cleaned and some fish were removed from the tanks to avoid crowding stress during the experiment. About 120 fish were stocked in each tank at 8 dpf, and 70 at 15 dpf, 33 at 35 dpf, 15 at 65 dpf. Water physicochemical parameters were measured one time a week by using Thermo scientific, ORION STRA A221 pH meter and UNIC 2800UV/VIS spectrophotometer. The dissolved oxygen and pH ranged 6.36–7.21 mg/l and 7.36–7.93, respectively. Ammonia nitrogen (NH_3_-N) and nitrite (NO_2_-N) never exceeded 0.11 mg/l and 0.3 mg/l, respectively. These parameters were well within acceptable levels for fish growth.

### Collection of gut, food, and water samples

The sampling procedures are shown in Figure S1. Fish were euthanized with MS-222 (100~200 mg/L). Body weight and standard length of the fish were measured during the experimental periods, as shown in Table S1. In brief, at 8 dpf, six gut samples were collected, and nine gut samples at subsequent each time point (at 18, 35, 65, and 125 dpf). Unable to distinguish various digestive regions, the entire gastrointestinal tract was collected at 8 and 18 dpf, and posterior gut was sampled at 35, 65, and 125 dpf. The collected samples were placed into a sterile tube and stored at −80°C until further sample processing.

Environmental microbiota samples were obtained at 8, 35, and 125 dpf. At each time point, one water sample and one food sample were collected from each tank. Approximate 250 ml of water in each tank was sampled and filtered through a 0.22 μm pore size filter. Filters cut into small pieces were put into sterile tubes and stored at −80°C for DNA extraction. Similarly, the prepared food for each tank was collected and stored in sterile tubes at −80°C after homogenization until further analysis.

### DNA extraction, sequencing preparation and processing

The bacterial DNA of gut and food samples were extracted by using QIAamp DNA Stool Mini Kit (Qiagen, Germany) according to the manufacturer's protocol. The DNA of water samples was isolated by using Mo PowerWater kits (MoBio, USA). An identical DNA concentration for all samples was prepared for amplification of the 16S rRNA gene spanning the V4-V5 region (515F, 5'-GTGCCAGCMGCCGCGGTAA-3' and 907R, 5'-CCGTCAATTCCTTTGAGTTT-3'). We added an adaptor and sample-unique DNA barcode of 12 base sequences at the 5' end of the PCR primers for sample identification. Amplifications were carried out in triplicate PCR mixtures (50 μl): 0.4 μM forward and reverse primers, 100 ng DNA template, 2.5U of GoTaq Flexi Polymerase (Promega, USA), 200 μM deoxynucleoside triphosphate (dNTP) and 2 mM MgCl_2_. Thermal cycling conditions for PCR were: 94°C for 5 min to denature the DNA, with a total of 25 cycles of 30 s at 94°C, 30 s at 55°C and 60 s at 72°C, and with a final extension of 5 min at 72°C. PCR products were purified by using Qiagen Gel Extraction Kit according to the manufacturer's instructions (Qiagen, Germany). The purified DNA was quantified using PicoGreen reagent and equal concentrations were pooled into one final pool. The pooled amplicons were sequenced with an Illumina HiSeq 2500 instrument (HiSeq Reagent Kit V.2, 500 cycles). Raw data are available on the NCBI BioProject under accession number PRJNA396633.

All raw sequence data analyses were done in Quantitative Insights Into Microbial Ecology (QIIME; version 1.8.0). Sequences were filtered to achieve high quality and assigned to respective samples. The processed sequences were clustered into Operational Taxonomic Units (OTUs) defined according to a value of similarity cutoff = 97% using the UCLUST algorithm. We then removed singleton OTUs (only one sequence read from the combined dataset) and classified taxonomically representative sequences into each OTU using Ribosomal Database Project (RDP) classifier with the Greengenes Database. In order to avoid the effects of sequencing depth on alpha and beta diversity, we performed rarefaction curve analysis and rarefied all samples to the lowest sequence reads for downstream analysis.

### Statistical analysis

Alpha and beta diversity in the data set were assessed in QIIME. For alpha diversity analysis, we calculated observed OTUs number, Shannon indices, Simpson indices, phylogenetic diversity whole tree (PD). For beta-diversity analysis, we used Bray-Curtis metric to evaluate bacterial community variations among all the samples and visualized in non-parameters multidimensional scaling analysis (NMDS) plots. In addition, we compared bacterial communities among gut, food and water samples collected at the same time points based on UniFrac distance metrics and plotted these samples using principal coordinate analysis (PCoA). Further, we used hierarchical agglomerative clustering with group average linking to identify group clustering. One-way analysis of variations (ANOVA) test was used to detect the significance of means in alpha diversity and dissimilarity among groups in IBM SPSS Statistics 19. Permutational multivariate analysis of variance (PERMANOVA) was performed to test the effects of host age on gut microbiota and to analyze bacterial community differences between the gut and surrounding environment. For pair-wise comparisons of gut bacterial communities, both PERMANOVA and analysis of similarity (ANOSIM) were used to test community differences based on Bray-Curtis metric and weighted UniFrac metric in PAST 3.0.

## Results

### Sampling depth and alpha diversity

A total of 2969724 V4–V5 16S rRNA sequence reads were obtained from 42 gut, six water and six food samples (on average: 49495 per sample; range: 26381-164795) after data quality control filtering and removal of primers, chimeras, and singletons. Alpha diversity of gut microbiota across host development is shown in Table 1. There was an increased trend in PD with host age (ANOVA, *p* = 0.064; regression model, *p* = 0.003). Moreover, three other alpha diversity indexes observed OTUs number, Shannon index and Simpson index increased linearly with age (ANOVA, *p* < 0.05; regression model, *p* < 0.01).

**Table 1:** Alpha diversity estimations of gut bacterial community in southern catfish at different host ages.

**Alpha diversity**	**Host age (dpf)**					**SEM**	***p***	**Liner model**	
	**8**	**18**	**35**	**65**	**125**			***p***	***r***
PD	12.99	13.26	15.33	15.64	16.99	0.51	0.064	0.003	0.443
OTUs	276.7	344.8	338.8	316.2	377.4	10	0.037	0.032	0.331
Shannon	1.81	2.27	2.55	2.71	2.75	0.1	0.044	0.003	0.449
Simpson	0.44	0.56	0.63	0.66	0.67	0.03	0.034	0.002	0.456

### Taxonomic composition of gut microbiota across different age stages

**Figure 1:**
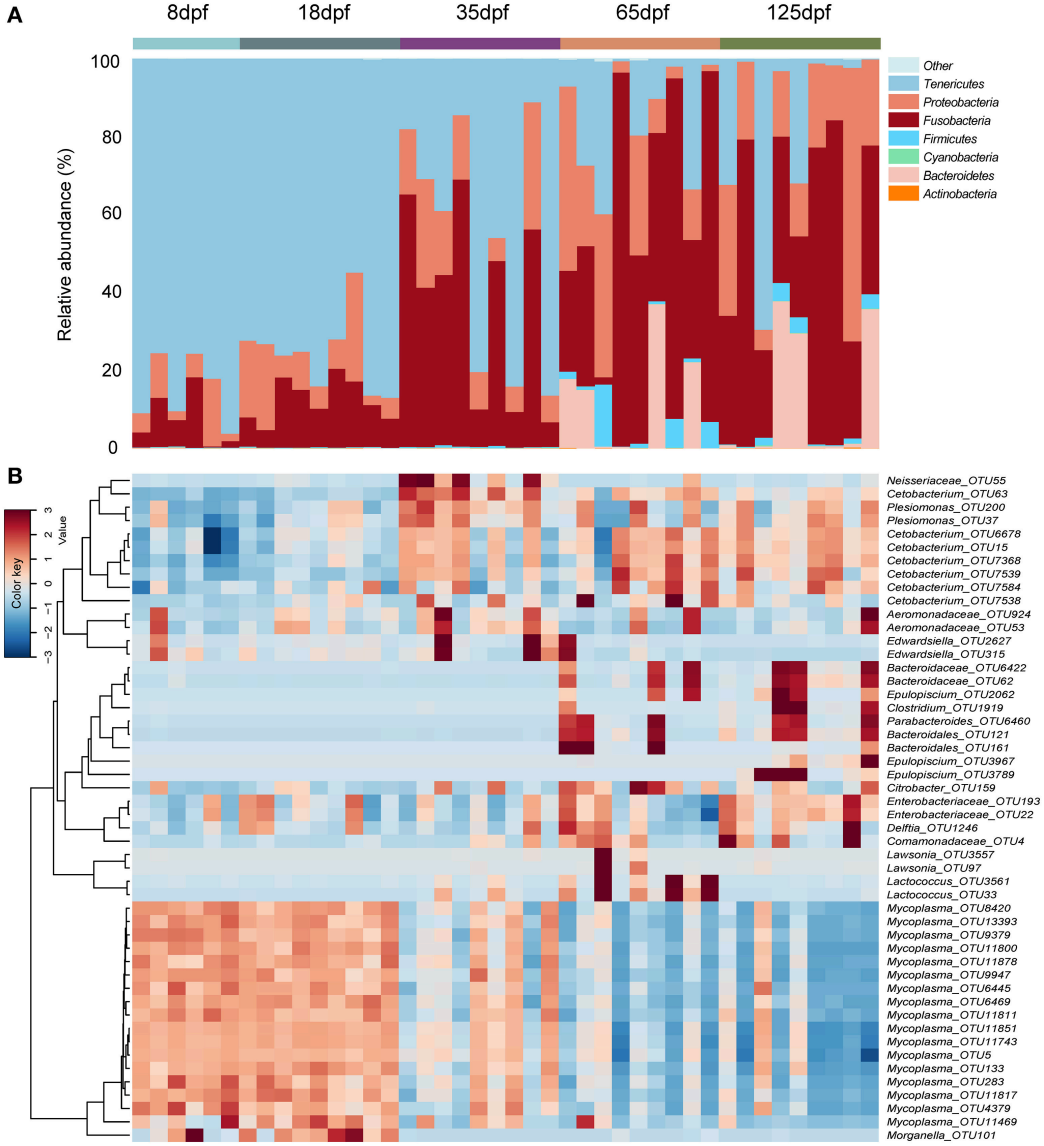
Compositions of bacterial community in the gut obtained from individual southern catfish at different host ages. **(A)** The microbiota is shown at the phylum level as bar graphs. **(B)** A heat map with the abundance of top 50 OTUs is represented. Bars are labeled by host age, with 8, 18, 35, 65, and 125 dpf representing southern catfish individuals collected at the ages 8, 18, 35, 65, and 125 dpf, respectively.

We observed eight phyla with more than 0.01% of relative abundance in the gut microbiota of southern catfish over the ontogeny. Of them, *Tenericute*s, *Fusobacteria, Proteobacteria*, and *Bacteroidetes* were the most abundant. The taxonomic composition of gut microbiota changed significantly across different age stages (Figure 1A and Figure S2). The phylum *Tenericute*s was the most abundant in individual samples at 8 and 18 dpf, with more than 75% of average abundance. The proportions decreased with host age (Kruskal-Wallis test, *p* < 0.001), while the *Fusobacteria* abundance significantly increased (*p* = 0.001), from 7.2% at 8 dpf to more than 45% at 65 and 125 dpf. Similarly, the abundance of *Proteobacteria* showed an increasing trend. Before 35 dpf, there were low values of the abundance of *Bacteroidetes*, which increased markedly at 65 and 125 dpf (*p* < 0.001). Of the observed phyla, which were mainly dominated by 21 genera with more than 0.1% of relative abundance at different stages (Figure S3). At 8 dpf *Mycoplasma* (*Tenericutes*) peaked in the gut and decreased with age (*p* < 0.001), meanwhile *Cetobacterium* (*Fusobacteria*) increased significantly (*p* = 0.001), toward the dominance at the late developmental stages. Several genera from *Proteobacteria* were dominated by unclassified *Enterobacteriaceae, Plesiomonas*, unclassified *Aeromonadaceae*, and *Morganella*.

### OTU diversity and dissimilarity analysis

**Figure 2:**
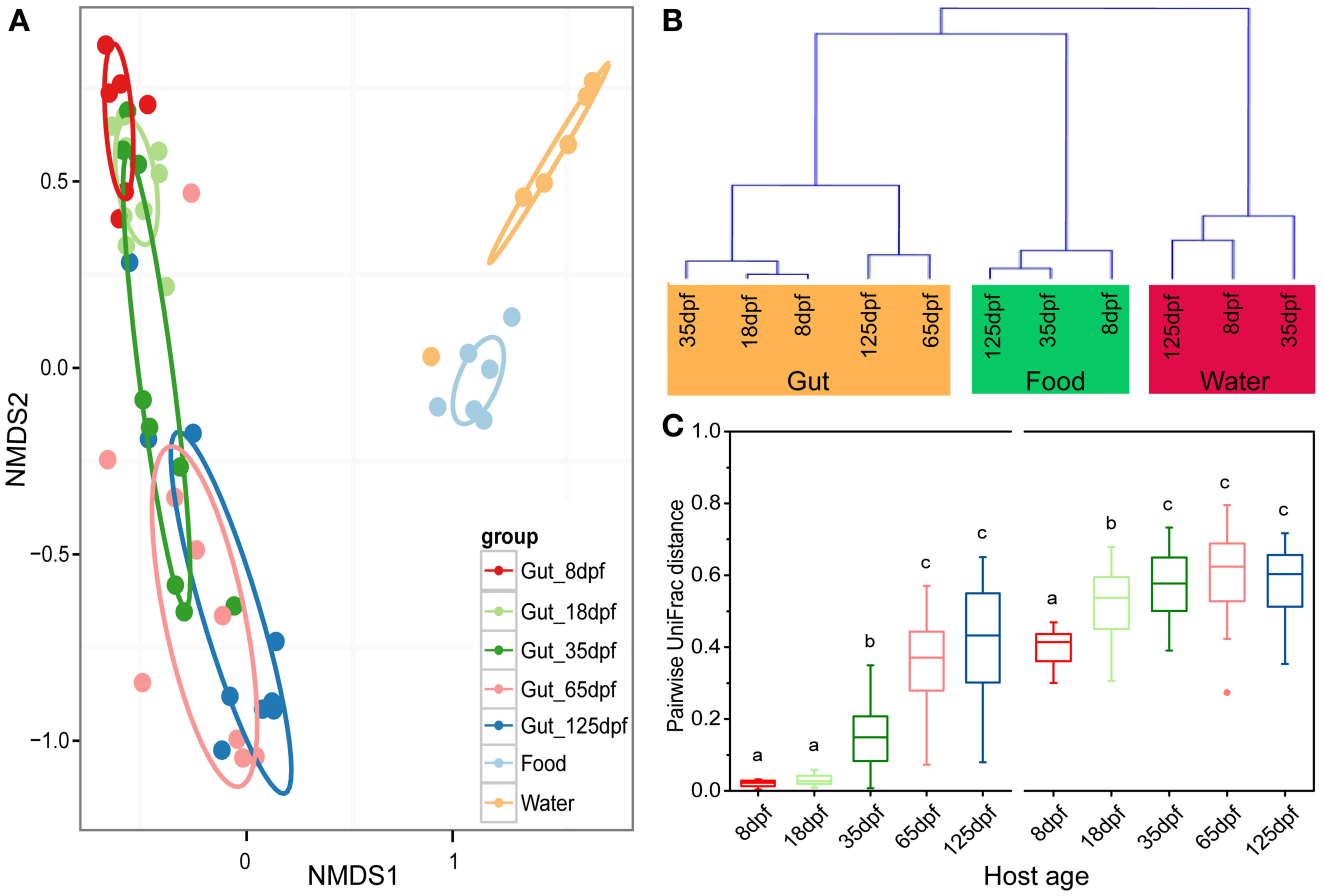
Partitions of bacterial community among southern catfish gut, food and rearing water at different host ages. **(A)** The microbiota is visualized by non-metric multidimensional scaling (NMDS) ordination based on Bray-Curtis distances. Food and water samples collected are not divided according to their collected time points. **(B)** Hierarchical agglomerative clustering with group average linking based on UniFrac distances for the gut, food and rearing water samples. **(C)** Pairwise UniFrac distances among the fish gut within each age group. Left based on weighted UniFrac distance; right based on unweighted UniFrac distance. The closer the value is to 1, the higher average dissimilarity within a group. Different letters above the bars indicate significance of microbial variations between age groups (*P* < 0.05).

The dominant OTUs in the gut showed temporal differences (Figure 1B). Microbial community comparisons at the OTU level visualized by NMDS based on Bray-Curtis distance metrics (Figure 2A) showed sample cluster according to age grouping (one-way PERMANOVA, *p* < 0.001). The individuals at 8 and 18 dpf were closer to each other than those at other developmental stages. Similarly, this trend was reflected in individuals at 65 and 125 dpf. The results of the pairwise comparison of PERMANOVA and ANOSIM consistently showed gut microbial assemblages were not different between 8 and 18 dpf, and between 65 and 125 dpf (Table 2). Moreover, gut microflora at 8 and 18 dpf significantly differed from those at 65 and 125 dpf (Figure 2B). Throughout the 5 stages of the ontogeny, the bacterial communities were similar in the early 2 stages which transited to the late 2 stages with microbial community reaching another similar and stable status. In addition, the average within-group dissimilarity based on UniFrac distance showed significant differences in microbial variations within individuals at the same developmental stages, and microbial variations increasing with host age (ANOVA, *p* < 0.001 for both distances, Figure 2C).

**Table 2:** Bray-Curtis distance and weighted UniFrac distance-based pairwise comparisons showing differences in the gut bacterial community in southern catfish at different host ages.

**Host age (dpf)**	**Bray-Curtis**		**Weighted UniFrac**	
	**PERMANOVA**	**ANOSIM**	**PERMANOVA**	**ANOSIM**
8 vs. 18	0.3235	0.1048	0.2492	0.6809
8 vs. 35	0.0491	0.0206	0.0349	0.1248
8 vs. 65	0.0002	0.0001	0.0003	0.0003
8 vs. 125	0.0005	0.0005	0.0023	0.0080
18 vs. 35	0.0111	0.0080	0.0184	0.0284
18 vs. 65	0.0001	0.0002	0.0001	0.0001
18 vs. 125	0.0003	0.0001	0.0002	0.0005
35 vs. 65	0.0733	0.0663	0.0003	0.0001
35 vs. 125	0.0690	0.0916	0.0071	0.0067
65 vs. 125	0.6800	0.7774	0.9385	0.6106

### Differences between gut and environment microbial communities

Gut samples and the corresponding environmental samples collected at 8, 35, and 125 dpf were used to compare the relationship between gut and environmental microbiota throughout southern catfish ontogeny. The total number of microbial OTUs in the gut was higher than that in the rearing water and food and increased in the gut over time (Figure S4). Similarly, the number of unique OTUs augmented in the gut, but not in the food and rearing water. Sample types affected alpha diversity (Table S2). Gut samples took on a lower alpha diversity than environmental samples in which all the diversity indexes were generally higher in the water than in the food at each sampling time point with an exception of PD. Further, PCoA showed a separation of the gut and environmental samples in both microbial structures and members at each time point (Figure S5). The results were consistent with the clustering link among groups of all samples throughout the host development (Figure 2B). Moreover, microbial communities in the water environment were more distantly separated from those in the fish gut than microbial communities in the food were (Figures 2A,B).

OTU classifications of microbial communities in the gut and environmental samples were performed at the genus level. Twenty-one most abundant taxa (on average, more than 1%) are shown in Table 3. *Cetobacterium* OTUs was the only shared abundant taxon by the gut, food and water samples. It was significantly more enriched in the gut (30.73%) than in the food (1.72%) and in the water (3.17%). The abundance of *Mycoplasma* was the highest in the gut (48.69%), but it was extremely low in the food (0.1%) and in the water (0.06%). *Treponema* consisted of 62.75% of microbiota in the food. However, its abundance accounted for only 0.01% in both the gut and rearing water. Other 13 taxa (more than 1%) in the water were either undetected or extremely low in abundance in the gut and food.

**Table 3:** The most common genera in the gut, food and rearing water of southern catfish at 8, 35 and 125 dpf^*^.

**OTUs classification (%)**	**Gut**				**Food**				**Water**			
	**8 dpf**	**35 dpf**	**125 dpf**	**AVG**	**8 dpf**	**35 dpf**	**125 dpf**	**AVG**	**8 dpf**	**35 dpf**	**125 dpf**	**AVG**
*Mycoplasma*	**85.09**	**45.01**	**15.96**	**48.69**	0.12	0.09	0.08	0.1	0.05	0.06	0.07	0.06
*Cetobacterium*	**7.2**	**38.88**	**46.1**	**30.73**	**3.58**	**2.69**	**1.72**	**2.66**	**0.2**	**7.65**	**1.65**	**3.17**
*Unclassified Enterobacteriaceae*	**4.93**	**4.95**	**17.88**	**9.25**	**23.66**	**17.76**	**12.89**	**18.1**	0.13	0.33	0.18	0.21
*Plesiomonas*	**0.84**	**5.44**	**3.67**	**3.32**	0.05	0.08	0.08	0.07	0.04	0.75	0.14	0.31
*Unclassified Bacteroidaceae*	**0.02**	**0**	**6.41**	**2.14**	0.01	0.01	0.02	0.01	0	0.16	0.01	0.06
*Bacteroidales^#^*	**0**	**0**	**4.31**	**1.44**	0	0	0	0	0	0.06	0	0.02
*Treponema*	0.01	0.01	0.01	0.01	**54.93**	**62.62**	**70.62**	**62.72**	0.01	0.01	0	0.01
*Lactococcus*	0.01	0.12	0.01	0.05	**2.53**	**1.96**	**1**	**1.83**	0.01	0.13	0.05	0.06
*Flavobacterium*	0.01	0	0.02	0.01	0.16	0.1	0.06	0.11	**41.98**	**17.66**	**24.72**	**28.12**
*Sediminibacterium*	0	0	0	0	0.01	0.01	0	0.01	**34.02**	**14.8**	**30.03**	**26.28**
*Limnohabitans*	0	0.01	0	0	0.03	0.02	0.02	0.02	**2.05**	**9.37**	**5.08**	**5.5**
*Comamonadaceae^#^*	0.01	0.02	0.02	0.02	0.17	0.21	0.17	0.18	**3.79**	**6.28**	**5.2**	**5.09**
*Clavibacter*	0	0	0	0	0.01	0	0	0	**4.22**	**2.03**	**5.29**	**3.85**
*Unclassified Comamonadaceae*	0.03	0.04	0.19	0.09	0.38	0.52	0.27	0.39	**1.27**	**6.63**	**3.14**	**3.68**
*Chryseobacterium*	0	0	0.01	0	0.23	0.17	0.07	0.16	**0.01**	**5.48**	**2.21**	**2.57**
*Flectobacillus*	0.01	0	0	0	0.01	0.02	0.01	0.01	**0**	**4.41**	**2.62**	**2.34**
*Rheinheimera*	0	0	0	0	0.01	0.01	0.01	0.01	**4.31**	**1.18**	**1.35**	**2.28**
*Pseudomonas*	0.01	0.02	0.05	0.03	0.12	0.14	0.1	0.12	**0.19**	**3.39**	**2.61**	**2.06**
*Novosphingobium*	0	0.02	0.01	0.01	0.04	0.06	0.01	0.04	**0.75**	**2.09**	**2.26**	**1.7**
*Unclassified Flavobacteriaceae*	0	0	0	0	0.05	0.04	0.01	0.03	**1.68**	**1.03**	**2**	**1.57**
*Rhodobacter*	0.01	0.04	0.01	0.02	0.08	0.19	0.12	0.13	**1.26**	**1.48**	**1.14**	**1.29**

* Average abundance of OTUs classifications (> 1%) in the gut, food or rearing water is shown. The means of relative abundance within each group and sample type (> 1%) are bold.

# *Genus is not identified and classified to other*.

## Discussion

Accumulated evidence has deciphered the importance of microbiota to host ontogenesis. In this study, we characterized gut microbial communities of southern catfish fed on a single food source across the development. As a consequence, we found a linearly increased alpha diversity and diverse bacterial communities at a 125-day developmental period under the consistent culture conditions. Furthermore, gut microbiota at different stages significantly differed from environmental microbiota. This suggests that host ontogeny shapes gut microbial development. We further conclude that microbial changes in ontogenesis can be independent of the effects of dietary shifts (Wong et al., 2015).

### Ontogenetic development of gut microbiota

Inter-individual variations were minimized by a batch of fertilized eggs from a pair of parents in this study. After the complete absorption of the yolk sac, southern catfish can feed on small aquatic animals at the first feeding. Thus, when southern catfish are supplied with the same food source, their gut microbial variations are relatively controllable and accessible. In our long-term feeding experiment, a gradually increased alpha diversity of gut microbiota in southern catfish was observed over time, demonstrating the direct effects of host age on gut microbial diversity. In several relatively short-term fish studies, microbial assemblages in the developing individuals resulted in an increase of the diversity (Ingerslev et al., 2014; Bakke et al., 2015). This allows us to speculate that the increased microbial diversity is independent of host species during the development. Two other studies aimed to explore the associations between developing hosts, showing the microbial diversity of significant decreases in zebrafish (Zac Stephens et al., 2016) and increases in gibel carp (Li et al., 2017). The resulting differences are possibly attributed to food shifts and different living environments at several critical stages throughout the lifespan of the hosts (Penders et al., 2006).

Gut ecological niche becomes more mature and is complex with host development so that the developmental differences can lead to changes in the community diversity (Zac Stephens et al., 2016). Compared to this study, more significant microbiota turnover was observed in Yan's et al. (2016) study on the same catfish but fed on boiled egg yolk in the first 3 days at the larval stage. The early dietary differences could be greatly responsible for microbial alterations. Several dominant OTUs persistently occurred in the gut of southern catfish. For example, *Mycoplasma* OTUs enriched in the southern catfish gut at the early stage in this study and was absent in the previous study (Yan et al., 2016). The *Mycoplasma* was detected to be abundant in both wild and lab fish (Holben et al., 2002; Lowrey et al., 2015). Additionally, widely distributed Atlantic salmon (*Salmo salar*) populations were also characterized by *Mycoplasmataceae* phylotypes (*Mycoplasma* OTUs especially) in the life-cycle stages (Llewellyn et al., 2016), suggesting the correlation between gut niche and developmental change. Notably, *Cetobacterium* OTUs increased at 18 and 35 dpf in this study, reaching the dominance at the late stages (Bledsoe et al., 2016; Zac Stephens et al., 2016). This is in line with the result observed in juvenile southern catfish fed on the same diet from 18 to 33 days post-hatching, but differing from that in the adult fish collected from a natural lake (Yan et al., 2016). The high abundance of *Cetobacterium* OTUs was consistently found in the gut of several freshwater fish as well as on-growing zebrafish at the late age (Zac Stephens et al., 2016; Zhang et al., 2017). The discrepancies among studies further underline the importance and necessity of host developmental context in studying host microbiota and their responses to external factors (Goffredi et al., 2014). Furthermore, cross-talk between host and diet at different stages should be addressed in further work (Wong et al., 2015).

Microbial communities of the gut in southern catfish underwent sequential changes with their development. At 8 and 18 dpf, within-group dissimilarity in the gut microbial communities was less diverse; it sharply increased before 35 dpf and stayed stable at the late stages. Evidently, the host was subjected to microbial successions during ontogenesis, potentially supporting the assumption that morphological development drives the changes in the microbiota and vice versa (Hooper et al., 2012; Olszak et al., 2012; Jiao et al., 2015). A parallel phenomenon has been observed in zebrafish (Zac Stephens et al., 2016) and other vertebrates (Jiao et al., 2015). Different gut microbial communities in southern catfish fed on an identical food at different developmental stages at least explain that gradual adaptation of host-microbiota which leads to the alterations in the prevailing microbiota in the gut.

### Correlations between gut and environmental microbiota

The fine-scale temporal sampling in the food and rearing water allowed us to evaluate microbial assembly relationships between the gut and external environment. The compositional relatedness analysis of microbial communities at multiple time points displayed that microbiota in the gut was differentiated from that in both the food and rearing water, suggesting gut microbial structure could not be simply explained by the environmental sources (Bakke et al., 2013; Wong et al., 2015; Yan et al., 2016). Despite different DNA extraction kits used in this study possibly leading to the differences in microbial communities between gut and environmental samples, a recent study showed that extraction method only explained small part of the community variation and did not result in inherent differences in taxonomic composition of microbial samples (Mackenzie et al., 2015). In the present study, some OTUs were shared by the gut, food and rearing water, but the environmental microbiota only accounted for relatively low abundances in the gut (Wong et al., 2015). Dominant microbiota in the catfish gut such as *Mycoplasma* was observed to be rather rare in the food and in the water (Llewellyn et al., 2016) and vice versa. For example, the highly abundant *Treponema* in the food and *Flavobacterium* in the water was poorly accumulated in the gut. On the contrary, Bledsoe et al. (2016) found OTU most abundant in the rearing water was dominant in the gut of channel catfish feeding endogenously at 3-day post-hatching. An introduction of the exogenous microbiota is likely to affect gut microbiota (Wu et al., 2013) through the competitions between microbiota and microbiota, and the interactions between host and microbiota. This process involves the influx of microbiota through fish feeding and excretion (Wu et al., 2013). Thus, the different abundance and enrichment of gut microbiota suggest the effects of early environmental exposure on initiation of host colonization.

The initial establishment of a nested gut microbial ecosystem is modulated by the ambient environment (Penders et al., 2006). The first exposure of fertilized eggs to water environment to some degree results in microbial colonization on their surface and unoccupied gut after hatching (Hansen and Olafsen, 1999) because microbiota in the aquatic environment can transfer freely from habitats to hosts. An early DGGE-based study of coho salmon (*Oncorhynchus kisutch*) reported that gut microbiota after first feeding was mainly from water and eggs epibiota (Romero and Navarrete, 2006). Surprisingly, recent work based on high-throughput sequencing showed that microbial compositions in late embryos poorly reflected microbial communities in surrounding water environment (Wilkins et al., 2015a,b), yet microbial communities in early embryos resembled those in the water environment (Wilkins et al., 2015a), potentially indicating that host selective pressures depend on different stages of developing embryos. At present, the lack of understanding of microbial associations between the environment and host at the continuous developments of fertilized eggs or embryogenesis is the case in many previous studies (Bakke et al., 2013, 2015; Ingerslev et al., 2014; Bledsoe et al., 2016). In addition to environmental effects, Wilkins et al. (2016) disclosed that salmonid fish egg-bacteria ecosystems were influenced by host genetics. Nevertheless, it is poorly understood that whether microbial responses to the hatching of fertilized eggs (from early embryos to late embryos) are consistent with their responses to individual growth after hatching (from early juveniles to adult) across the development, and whether the responses can be correlated with environmental microbiota at the initial stage of colonization. Samples before 8 dpf in this study were not collected, resulting in a failure in performing earlier microbial comparisons between the fish eggs and water. However, the dissimilarities of community structure between the developmental gut and water were higher than those between the gut and food, suggesting that microbiota in water has probably less effect on gut microbial community than that on food since the host begins with exogenous feeding (Llewellyn et al., 2014; Li et al., 2017).

In summary, we have demonstrated that host age was an important factor influencing the gut microbial community in southern catfish. An increase in gut microbial diversity occurred during the ontogenesis after exogenous feeding. The influences of host age on microbial community disappeared at the late stages under the controlled exogenous conditions such as diets that are the possible source of microbial community variation, indicating a vital role of early host development in shaping microbial occurrence in the gut. By continuous comparisons between host and environment, we found that gut microbial structure did not reflect environmental microbiota. These results suggest gut microbial changes could result from the enhanced adaptation to gut development. Further work can concentrate especially on the role of embryo hosts and environmental microbiota during the embryogenesis, and disentangle the effects of the initial establishment of an embryos-associated community on late individual microbial development and life history of the host. Quantifying surface microbiota from eggs to embryos stages, and to individual development associated with different sources of environmental microbiota will allow us to fully characterize temporal microbial dynamics of fish development, and to reveal how the early exposure of ontogeny affects late microbial colonization and persistence. It will provide an insight into the host selection of gut microbiota and microbial modulations in teleosts.

## Author contributions

DL and ZZ designed the experiment. ZZ, WX, and MR conducted the experiment. ZZ, DL, and WX analyzed the data. ZZ, DL, MR, RT, and LL wrote the manuscript.

### Conflict of interest statement

The authors declare that the research was conducted in the absence of any commercial or financial relationships that could be construed as a potential conflict of interest.
