# Supplementary material for: Host Age Affects the Development of Southern Catfish Gut Bacterial Community Divergent From That in the Food and Rearing Water
Source: Front Microbiol. 2018 Mar 20;9:495. doi: 10.3389/fmicb.2018.00495 (PMC5869207; doi:10.3389/fmicb.2018.00495)
Supplement: Supplementary file 1 [file Presentation1.PDF]

Host age affects the development of southern catfish gut bacterial community divergent from that in the food and rearing water

***Zhimin Zhang*[1,2]*, Dapeng Li*[1,2*]*, Mohamed M. Refaey*[1,2,3]*, Weitong Xu*[1,2]*, Rong Tang*[1,2]*, Li Li*[1,2]**

[1] College of Fisheries, Huazhong Agricultural University, Wuhan, P.R. China, [2] Hubei Provincial Engineering Laboratory for Pond Aquaculture, Wuhan, P.R. China, [3] Department of Animal Production, Faculty of Agriculture, Mansoura University, Al-Mansoura, Egypt

Running title: Host age affects gut microbial development

[*]Address correspondence to Dapeng Li, ldp@mail.hzau.edu.cn

Table S1. Body weight and length of southern catfish sampled at different host ages.

Growth index	Host age				
	8dpf	18dpf	35dpf	65dpf	125dpf
Body weight (g)	0.101±0.01	1.42±0.08	5.52±0.41	33.11±2.11	119.56±7.92
Body length (cm)	2.02±0.07	4.06±0.2	6.91±0.50	15.87±0.92	25.61±1.11

Table S2. Comparisons of alpha diversity of bacterial community among southern catfish gut, food and rearing water at 8, 35 and 125 dpf.

Alpha diversity	8 dpf			35 dpf			125 dpf		
	Gut	Food	Water	Gut	Food	Water	Gut	Food	Water
PD	12.99	31.92	20.33	15.33	34.22	30.05	16.99	31.30	26.28
OTUs	276.7	477.5	487.5	338.8	562.0	707.0	377.4	494.5	631.0
Shannon	1.81	3.44	3.55	2.55	3.31	5.59	2.75	3.00	4.78
Simpson	0.44	0.73	0.79	0.63	0.69	0.94	0.67	0.62	0.89

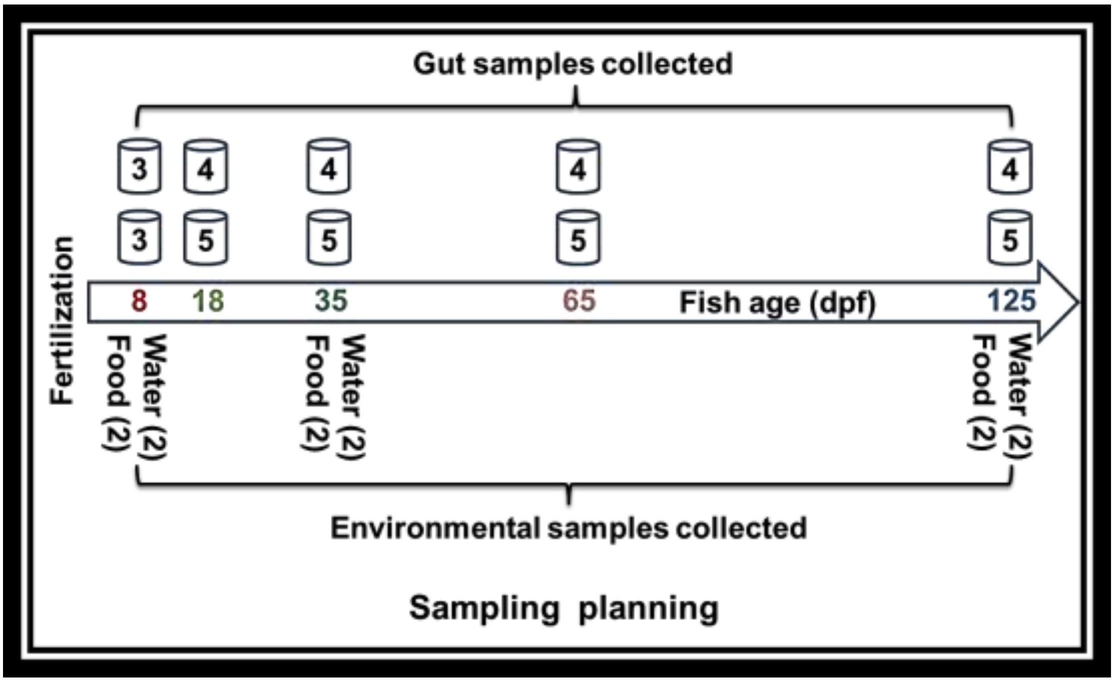

Figure S1. Experimental design for sample collections of southern catfish gut, food and rearing water. The numbers in arrows represent fish age. The numbers above arrows represent the collected gut sample numbers from each tank at different host ages. The numbers below arrows represent the collected environmental sample numbers from two tanks at the corresponding host age.

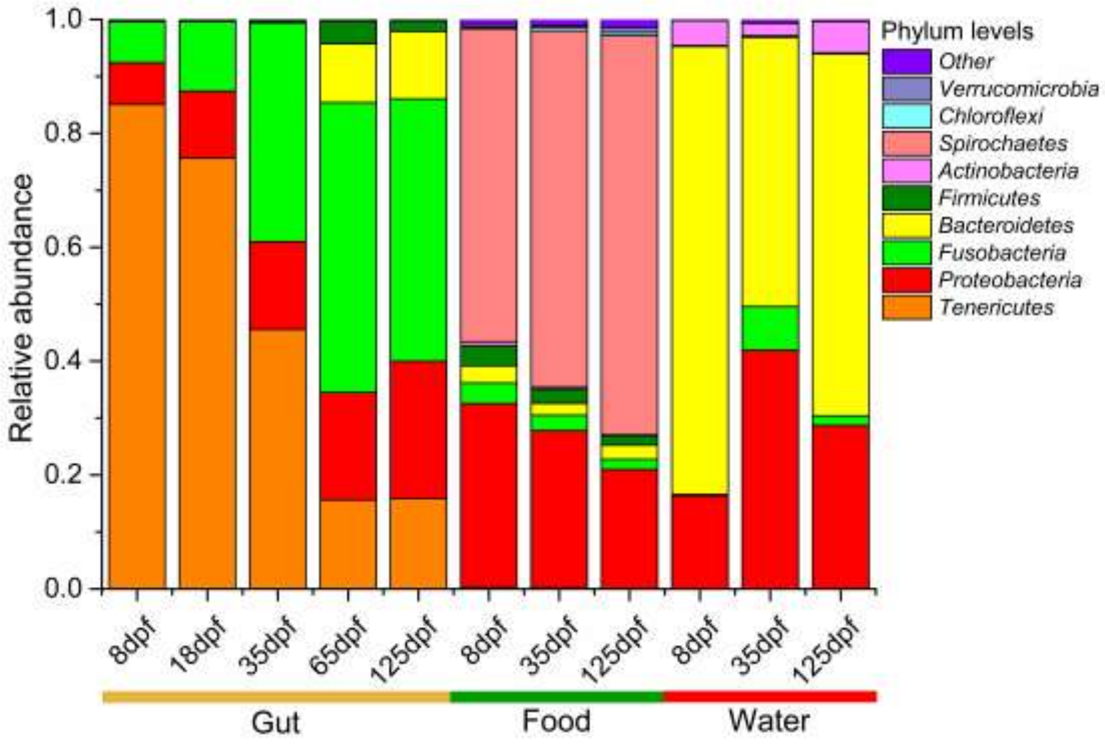

Figure S2. Bacterial compositions of gut, food and rearing water at the phylum levels at different host ages.

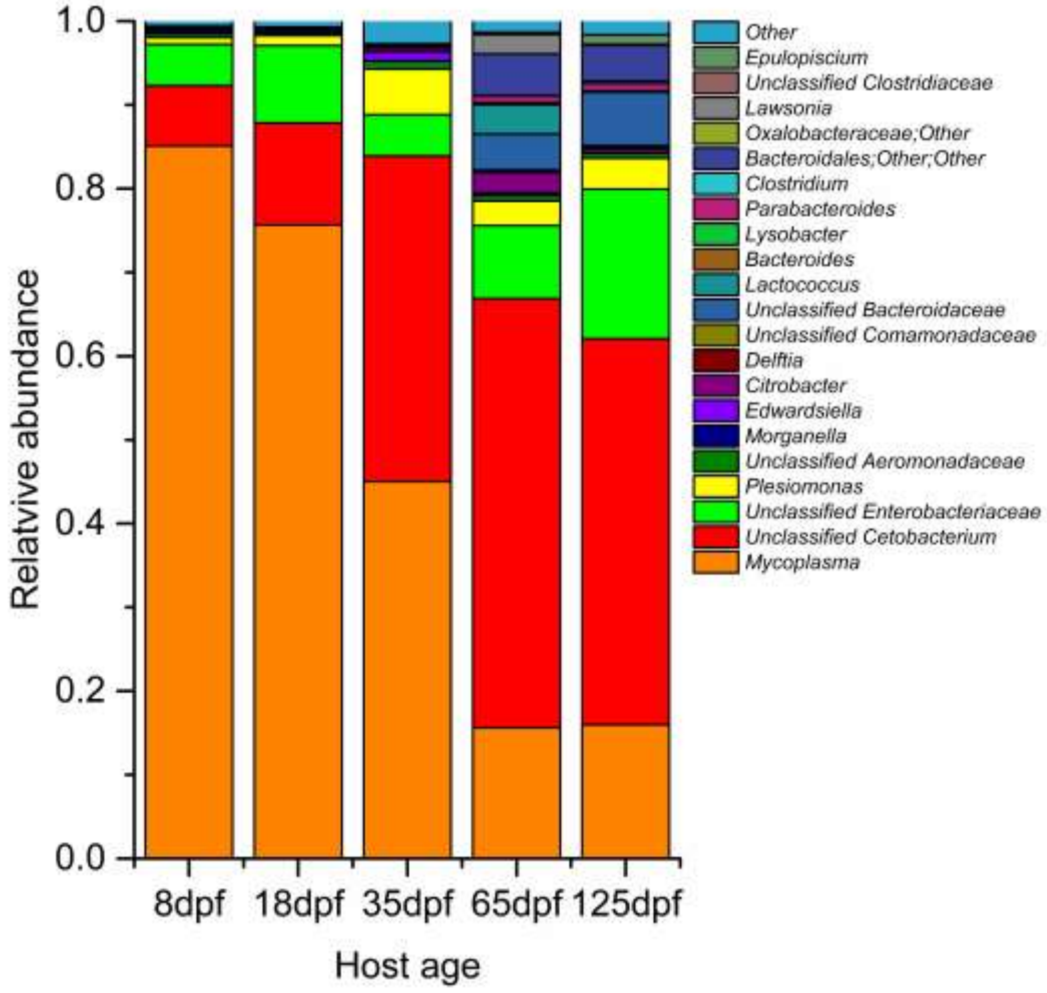

Figure S3. Bacterial compositions of southern catfish gut at the genus levels at different host ages.

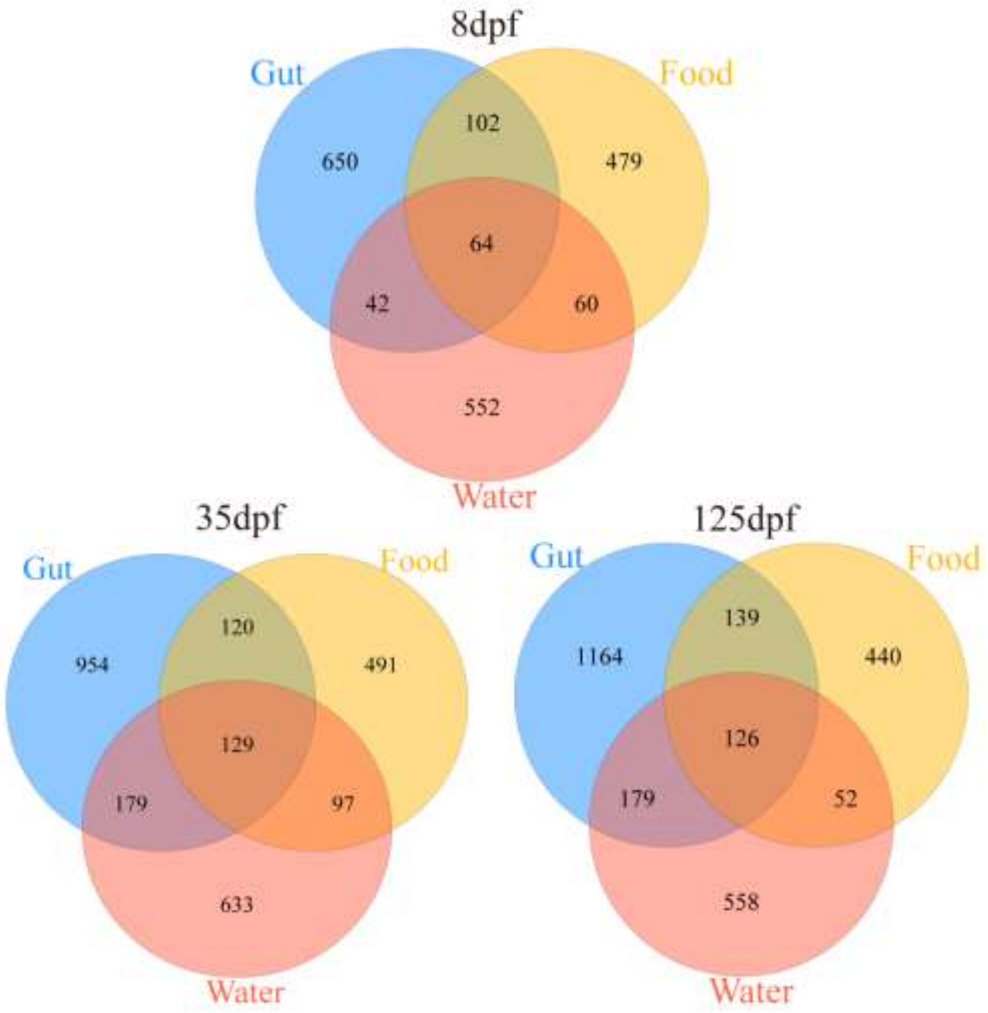

Figure S4. Unique and shared OTUs numbers among southern catfish gut, food and rearing water at 8, 35 and 125 dpf.

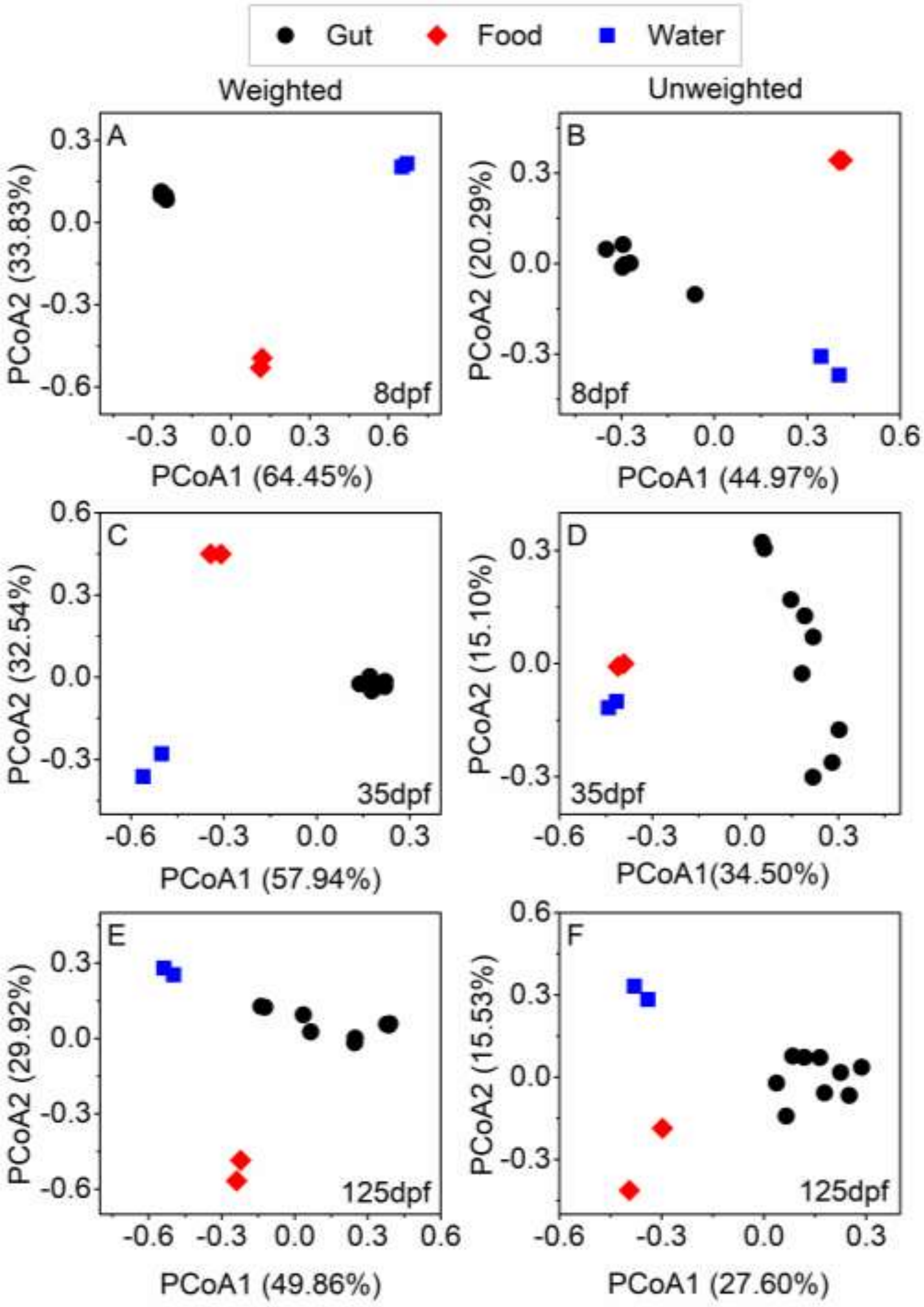

Figure S5. Principal coordinate analysis plot based on unweighted and weight UniFrac distance for bacterial community comparisons among southern catfish gut, food and rearing water at 8, 35 or 125 dpf.
